# Creating a caregiver education program with chinese caregivers of autistic children: A community engaged process

**DOI:** 10.1371/journal.pone.0355749

**Published:** 2026-08-13

**Authors:** Yi Yang, Qichao Pan, Yi Xin, Xuejing Liu, Hongrui Qiu, Moon Young Savana Bak, Ana D. Dueñas, Amber M. Reilly, Natalie G. Dumas

**Affiliations:** 1 Department of Special Education, School of Education Sciences, Guangdong Provincial Key Laboratory for the Education and Development of Children with Special Needs, Lingnan Normal University, Zhanjiang, Guangdong, China; 2 Department of Special Education, University of New Mexico, Albuquerque, New Mexico, United States of America; 3 Department of Special Education & Counseling, The Education University of Hong Kong, Hong Kong, China; 4 Counseling, Educational Psychology & Special Education Department, Michigan State University, East Lansing, Michigan, United States of America; 5 Department of Mental Health, Johns Hopkins University, Baltimore, Maryland, United States of America; 6 Department of Educational Psychology, University of Minnesota, Minneapolis, Minnesota, United States of America; 7 Department of Special Education, University of Washington, Seattle, Washington, United States of America; Nagoya University: Nagoya Daigaku, JAPAN

## Abstract

Despite a growing body of research highlighting the advantages of caregiver education for both autistic individuals and their caregivers, little is known about what Chinese caregivers of autistic children may want and need within caregiver education. In this qualitative study, we invited 14 Chinese caregivers of autistic children to share their needs and perspectives on the content and delivery of a caregiver education program. Specifically, we conducted two focus groups. The first focus group asked caregivers about the desired content of caregiver education. The second focus group asked for feedback on two prototype videos created based on the first focus group. We used reflexive thematic analysis, revealing three emergent themes regarding content and two themes regarding the delivery method. The study results show that the content and modality of online caregiver education programs should be created by solicitating intended user input throughout the development process.

## Introduction

A recent large-scale regional study in China reported a national estimated prevalence of autism at 0.70% among children aged 6–12 years [1]. Although there is an increasing public awareness of autism [2] and ongoing efforts to establish autism-specific policies and laws by the Chinese government [3], research consistently indicates a substantial demand for support among Chinese families with autistic children [4]. Similar to caregivers of autistic children in many other countries, Chinese caregivers actively participate in providing appropriate and effective services to their autistic children [2,5]. However, unlike their counterparts in countries with a more established autism support system, many Chinese caregivers may serve dual roles as primary caregivers and primary service providers to their autistic children by implementing interventions at home [6]. Even with Chinese caregivers actively assuming roles as intervention providers for their autistic children, obstacles persist, including limited free and trustworthy autism resources [7]; societal stigma [8]; and the financial burden of services [4].

Caregiver education can increase support for families with autistic children and mitigate disparities in autism services [9,10]. Studies have systematically assessed the effectiveness of caregiver education, demonstrating their positive impact not only on child outcomes but also on the enhancement of parenting skills, child-parent relationships, and the mental well-being of caregivers [11,12]. Caregiver education typically provides information regarding autism, child development, and evidence-based practices; it also trains caregivers to implement specific interventions such as positive reinforcement, modeling, and prompting [13]. Caregiver education can be delivered as a one-time service, a manualized intervention for a specific intervention, or a comprehensive education following a pre-determined curriculum [14]. An example of a one-time service would be a one-day presentation on toilet training. An example of a manualized intervention may involve enrolling participants for a particular duration (e.g., 8 weeks) to train and implement a specific intervention. An example of a comprehensive education would be the online Autism Focused Intervention Resources and Modules (AFIRM; https://afirm.fpg.unc.edu/).

If caregiver education were to be utilized to its full potential to mitigate service disparities, accessibility, sustainability, and acceptance decided by caregivers would be critical [15]. However, in much of the extant literature investigating the effectiveness of caregiver education, caregivers are typically relegated to the role of passive recipients (i.e., study participants) rather than active contributors, and crucial aspects of caregiver education programs, such as content, modality, duration, and accessibility are determined by researchers [14]. This can result in caregiver education that requires participants to navigate childcare, employment flexibility, and easy access to the study location or technology, and may not apply to many communities [15,16]. Furthermore, research emphasizes developing and utilizing culturally appropriate education materials for caregivers from diverse backgrounds [17].

A community-engaged approach to caregiver education can reduce participation barriers and increase caregivers’ acceptability and involvement. For example, Milgramm and colleagues implemented a caregiver education program offered on a rolling-enrollment basis, community-based group of participants [18]. Milgramm and colleagues also designed the education program’s content using a needs assessment survey collected from caregivers of autistic children in the community. After the caregiver education was concluded, the results revealed that all dependent measures, including parent self-efficacy, knowledge, and satisfaction, increased [18]. In China, Fang and colleagues (2022) used interviews and focus groups to explore the experiences and acceptability of 14 Chinese caregivers of autistic children aged 3–6 years *after* they participated in a short-term intensive parent education program and found that caregivers were eager to identify areas for improvement regarding program content (e.g., additional training targeting other family members) and delivery methods (e.g., practitioner modeling) [19]. However, to the best of our knowledge, no study in China has included caregivers in the pre-development stages of a caregiver education program.

Therefore, this preliminary study aimed to address this gap by gathering perspectives and insights into the specific needs and preferences of Chinese caregivers of autistic children in the *pre-development stages* of creating a caregiver education program using a community-engaged approach. We conducted two stages of focus group discussions with Chinese caregivers of autistic children: 1) before developing the caregiver education content using an interview guide and 2) after we had built two prototype video modules based on the first focus group discussions. Specifically, we asked the following research questions: What *content* do Chinese caregivers of autistic children want in a caregiver education program? What *presentation method* do Chinese caregivers of autistic children want in a caregiver education program?

## Method

### Design

The study employed a qualitative design, utilizing focus group discussions as the data collection strategy. This decision was grounded in the participants’ shared experiences of parenting autistic children and the research goal to elicit a deeper and richer understanding through the social interactions within the group [20].

### Participants

#### Recruitment.

Participants for the study were recruited through recruitment emails distributed to autism intervention service providers and caregiver organizations in mid-to-small-sized cities in mainland China. To be eligible for participation, caregivers had to meet the following criteria: 1) identify as Chinese, 2) serve as the primary caregiver for at least one autistic child under the age of six, and 3) express willingness to engage in two one-hour focus group discussions (i.e., Topic 1, Topic 2). Topic 1 focused on the content participants would like to see in a comprehensive caregiver education program. In contrast, Topic 2 was focused on the delivery method the participants would like for a comprehensive caregiver education program. The age criteria for children were restricted to those under six years old, aligning with the predominant focus of available services in China on early childhood [4] and assisting caregivers in preparing their children for school readiness [21].

#### Demographic information.

In total, 14 caregivers of autistic children were recruited for the study. Written consent was obtained from all participants, and the research protocol was approved by the Institutional Review Board. Among the participants, 11 identified as mothers, and three identified as fathers of autistic children. The caregivers’ ages ranged from 31 to 50, with 85.7% (n = 12) falling within the 31–40 age group. Table 1 provides characteristics of caregivers and their autistic child(ren).

**Table 1:** Characteristics of participants.

Caregiver						Child			
	Gender	Age (yrs)	Education Level	Topic 1 Participation	Topic 2 Participation	Age_yrs (interview)	Age_yrs (diagnosis)	Intervention Duration	Intervention Intensity
P01	M	31-40	College	Group 1A	Group 2B	5	3	2 yrs	High
P02	F	31-40	College	Group 1A	Group 2B	3	2	1 yrs	Moderate
P03	F	31-40	College	Group 1A	Group 2A	2	1	3 mos	Low
P04	F	31-40	NR	Group 1B	N/A	4	2	2 yrs	Low
P05	F	31-40	College	Group 1B	Group 2B	4	3	1 mos	Low
P06	M	31-40	College	Group 1B	Group 2B	4	3	1 mos	Low
P07	F	31-40	College	Group 1B	N/A	6	1	3 yrs	Low
P08	F	31-40	College	Group 1B	Group 2A	5	3	1 yrs	Moderate
P09	F	41-50	Graduate School	Group 1B	N/A	3	NR	2 yrs	Low
P10	F	31-40	College	Group 1C	N/A	5	3	1 yrs	Moderate
P11	F	31-40	College	Group 1C	N/A	4	2	1yrs	High
P12	M	41-50	College	Group 1C	Group 2B	5	2	2 yrs	High
P13	F	31-40	College	Group 1C	Group 2A	5	3	2 yrs	High
P14	F	31-40	College	Group 1C	Group 2A	4	3	5 mos	Moderate

*Note.* F = female; M = male. NR = Not reported. High = high intensity of intervention (30–40 hours every week); Moderate = moderate intensity of intervention (20–30 hours every week); Low = low intensity of intervention (0–10 hours every week). yrs = years; mos = months. N/A = not applicable.

In the Topic 1 focus group discussions, all 14 caregivers actively participated, organized into three groups (i.e., 1A, 1B, 1C) based on their availability for the online meetings. In the Topic 2 focus group discussions conducted two months later, not all original participants were able to commit to the online meeting due to various personal reasons, many of which were childcare and family-care obligations in the context of several severe COVID-19 outbreaks after the early COVID restrictions had been lifted in China. As such, *nine* out of the original 14 participants were divided into two groups (i.e., 2A, 2B) and joined the Topic 2 focus group discussion. Detailed participation information is provided in Table 1.

### Materials

#### Survey.

Before the focus group discussions, participants were asked to complete an online demographic questionnaire and the Family Empowerment Scale [22]. The FES is a 34-item self-report measure used to assess families’ perceived empowerment. It includes nine questions on system advocacy, 11 questions on knowledge, eight questions on competency, and six questions on self-efficacy [22].

#### Topic guide for focus group.

Following the standard focus group methodology, two distinct topic guides were developed based on different research questions for two focus group discussions.

**Topic 1 Focus Group.** To guide the discussions of Topic 1, we utilized a topic guide that included basic introductory information on child development, early identification, and diagnostic criteria adapted from Autism Speaks (https://www.autismspeaks.org/) and basic behavioral intervention information adapted from the Registered Behavior Technician (RBT) Task List developed by the Behavior Analyst Certification Board [23]. The introductory information from Autism Speaks on child development, early identification, and diagnostic criteria was selected because it is typically the first information caregivers receive following their child’s autism diagnosis. The RBT Task List was selected because it contains entry-level information for delivering evidence-based behavioral support to autistic individuals. After the topic guide was decided on and written in English, the first author and the second author—trained in special education with a focus on autism and with experience as behavior analysts serving autistic children in China—undertook translations and cultural adaptations to the topic guide. Items that were irrelevant to China (e.g., American Academy of Pediatrics’ Well-Child Care visit) and caregivers (e.g., professional conduct and supervision) were removed, and additional items that caregivers would be interested in based on the first authors’ continuing service experience in China (e.g., naturalistic developmental behavioral interventions) were added. Participants received the topic guide via email a week before the Topic 1 focus group discussions to review. The topic guide is provided as Supporting Information 1 (i.e., S1 File).

**Creation of Prototype Video Modules.** After Topic 1 focus group discussions were completed, we analyzed the results and created two prototype education videos based on participant feedback. The first video (i.e., Video 1; 6 min 30 s) provided an overview of how behavioral principles impact behavior, introducing applied behavior analysis (ABA) and its dimensions. This video aimed to empower caregivers by helping them understand the foundational concepts of behavior and the importance of analyzing behavior to better understand their children based on the existing and growing popularity of ABA-based services in China [4]. The second video (i.e., Video 2; 9 min 30 s) covered the antecedents and consequences of behavior, serving as an introductory resource for caregivers to grasp the basics of functional behavior assessments. The authors first created video content as a storyboard outline with images and accompanying scripts. Then, both storyboards were produced by a professional video production company, and a combination of lectures by the first author, with presentation slides and videos with animated characters modeling examples, were utilized.

**Topic 2 Focus Group.** Two months after the conclusion of the Topic 1 focus group, participants were invited to participate in the Topic 2 focus group discussions. Videos 1 and 2 served as topic guides for the Topic 2 focus group discussion and were sent to participants one week before the focus group discussions. The participants were asked to watch the videos before their focus group meeting. Links to videos are available upon request.

### Procedure

#### Focus group discussion.

**Topic 1 Focus Group.** Fourteen participants were divided into three groups based on availability and met via online meeting software. Group 1A comprised three participants, Group 1B had six participants, and Group 1C included five. The focus group discussions lasted approximately 60 minutes for each group—all facilitated by the first and second authors. At the beginning of each group meeting, a facilitator outlined the research objective and general rules of group discussions. Participants were then allowed to introduce themselves only by their last name to maintain confidentiality. The facilitators (i.e., the first and second author) screen-shared the topic guide after the introductions. They introduced the items to initiate the discussion regarding the content of proposed caregiver education programs.

**Topic 2 Focus Group.** Nine out of the original 14 participants were able to join Topic 2 focus group discussions, which were divided into two groups (i.e., four in Group 2A and five in Group 2B). The first author facilitated the Topic 2 focus group discussions, which followed the same format as Topic 1 (i.e., introductions). Participants were encouraged to share their feedback on the two videos and offer suggestions.

#### Data analysis.

We used reflexive thematic analysis [24,25] to analyze the focus group discussions. This analytical approach was selected to facilitate identifying and exploring recurring themes within the discussion data and offer a nuanced understanding of the caregivers’ perspectives and needs regarding caregiver education for autistic children in the Chinese context. Both focus group discussions (i.e., Topic 1 and 2) were recorded and transcribed using online conferencing software. Two research assistants who are Chinese and fluent in Chinese checked the transcribed transcripts for accuracy. Four authors (MASKED FOR PEER REVIEW) who identify as Chinese and are fluent in Chinese participated in the analysis. The reflexive thematic analysis process in this study involves six phases: (1) *Familiarization with the data*: each author took individual time to thoroughly read the transcripts and take notes to understand the content and context of the data; (2) *Systematic data coding*: the authors independently coded the entire transcripts, identifying key ideas of data that might be relevant to address research questions; (3) *Generating initial themes*: the authors met together to collectively examined, compared, and discussed individual codes to identify connections and group them into candidate themes. This process continued until consensus was reached on the initial themes; (4) *Developing and reviewing themes*: the authors met again to revisit the initial themes, checking against the coded data to ensure accuracy and alignment. Adjustments to themes, such as splitting, combining, or discarding themes, were made during this stage until the team reached an agreement; (5) *Refining, defining, and naming themes*: the authors determined the scope and focus of each theme and finalized theme names and definitions through collaborative discussions; (6) *Writing the report*: themes were synthesized with supporting data extracts integrated to present the findings of this study. All analyses and discussions were conducted in Chinese. The second author translated the transcripts and results into English for dissemination purposes, and the third author conducted accuracy checks for these translations. Positionality statements are provided in as Supporting Information 2 (i.e., S2 File).

## Results

### Survey

The mean total score for fourteen participants of this study on the FES was 124.6 out of 170, with a range of 104–150. For the subscales of the FES, the mean score was 30 (Range: 23–34; SD = 3.40) out of 45 for the System-advocacy subscale, 38 (Range: 31–51; SD = 5.08) out of 55 for the Knowledge subscale, 30 (Range: 24–38; SD = 4.16) out of 40 for the Competency subscale, and 24 (Range: 19–29; SD = 2.97) out of 45 for the Self-efficacy subscale, indicating high family empowerment in our participants. In addition, responses in the Self-efficacy subscale have the least variability, indicating greater consistency among participants, while the responses in the Knowledge subscale showed the highest variability, suggesting a wider spread of scores within that category.

### Focus group discussions

Three themes were identified from the Topic 1 focus group: 1) understanding autism from cause to intervention, 2) learning behavioral principles and techniques, and 3) support for caregiver mental health. Two themes were identified from the Topic 2 focus group: considerations for accessible language and cognitive load and the importance of case examples and modeling. The original quotes (i.e., Chinese) are presented as Supporting Information 3 (i.e., S3 File). The definition of themes and exemplar quotes are provided in Table 2.

**Table 2:** Themes and Exemplary Quotes.

Theme	Definition	Exemplary Quote
**Content**		
**Autism: From Cause to Intervention**	Caregiver training programs should cover content in addressing the causes of autism, the characteristics of autism, and how it differs from other developmental disabilities. It should also provide guidance on how to utilize evidence-based interventions to support the needs of autistic children following their diagnosis.	*We first want to know the cause of these children’s symptoms. In other words, maybe the priority is to find out the reason. Figuring out what is affecting the child and what scientific methods are appropriate for intervention. I think this might be a better way for families and parents* (P03, 1A).
**Behavioral Principles and Techniques**	Caregiver training programs should cover content in basic behavioral principles to better understand the behaviors of autistic children. It should also offer practical applications of behavioral techniques for use in home and community settings.	*I felt I understood the concepts but did not know how to use them practically. Like the generalization, I think it is hard, and I am unsure if I can help my child generalize one skill to daily living…. I am confused about to which degree the training goal was reached; I remember it was 80% accuracy, and then we could say that the target had been reached. However, there is still a difference when applying those skills to daily life* (P09, 1B).
**Caregiver Mental Health Support**	Caregiver training programs should cover content in supporting caregivers in accepting the autism diagnosis and offers strategies for self-care to reduce anxiety related to autism and child development. It should also emphasize the importance of connecting with other caregivers for support.	*As a parent who has gone through this, I feel that you need to add something about how to relieve the anxiety of parents. Although I am at a different stage now, if the child has not been progressing, the anxiety of parents is a lot…Whenever our children have difficulties learning, parents of these children, including the parents around me, will be generally anxious and even have anxiety bursts. In most situations, such anxiety will affect the child. So, if you are going to teach parents, my suggestion is to add a module about helping parents relieve anxiety as well as stop self-blame* (P01, 1A).
**Theme**	**Definition**	**Exemplary Quote**
**Format**		
**Accessible Language and Cognitive Load**	Caregiver training programs should use simple, accessible language, minimizing jargon and acronyms. It should also allow for individualized training duration, catering to caregivers at different stages and giving them the option to choose their preferred cognitive load during training.	*When I read books or listen to lectures, some terms are difficult to understand. I need to spend more time processing them and making them more direct. I am unsure whether other parents would have the same feeling* (P04, 1B).
**Case Examples and Modeling**	Caregiver training programs should include sufficient case examples to explain concepts, using modeling strategies to demonstrate intervention techniques in settings that closely resemble real-life situations.	*As a new parent like me, everything is good when my child is in the intervention center with teachers, but I really cannot cope with my children’s needs at home. Part of the reason is my limited ability, so if there is a video, parents can just learn to follow the modeling to teach kids* (P03, 2A).

#### Topic 1: What Content Would You Like to See in a Caregiver Education Program?.

**Theme 1 – Autism: From Cause to Intervention.** From cause to intervention, autism-related knowledge emerged as the participants’ primary thematic focus for a caregiver education program. Many participants expressed confusion upon initially encountering information about autism or upon receiving an autism diagnosis for their children, citing a scarcity of trustworthy resources. Specifically, caregivers shared that distinguishing between autism and other developmental disorders or psychiatric conditions posed a challenge, especially when the next step was to identify appropriate support and service. Furthermore, caregivers expressed that it was particularly challenging to understand the relationship between the characteristics exhibited by their children and the diagnosis of autism, especially in instances where medical professionals were unable to provide a clear and comprehensive explanation of autism and its possible heterogeneity in individual expression.

*We first want to know the cause of these children’s symptoms. In other words, maybe the priority is to find out the reason. Figuring out what is affecting the child and what scientific methods are appropriate for intervention. I think this might be a better way for families and parents* (P03, 1A).

*My child was diagnosed at the age of three, but the diagnosis at first was very unclear. We spent two to three hours on the diagnostic process, filling in measures, etc. The doctor only took 10 minutes to observe my child and said that my child is autistic and needs interventions. I was very confused and skeptical about the diagnosis – how the doctor could only use a few minutes to diagnose my child. After I went back home, I looked up the information about autism, did observations on my child, and finally accepted this diagnosis* (P13, 1C).

Incorporating evidence-based practices for autistic individuals within the framework of educational programs was another important component proposed by our participants. Some caregivers recounted their negative experiences with interventions for their autistic children that lacked robust scientific support (e.g., acupuncture). Notably, participants shared that even medical professionals from hospitals sometimes recommended treatments with insufficient empirical evidence. Participants strongly expressed the need for introducing comprehensive information about research-based interventions for autistic children. In addition, participants emphasized the need to empower caregivers with knowledge to discern the veracity of given information, thereby fostering a more informed decision-making process.

*Parents who just received their child’s diagnosis do take the wrong path, and I think the first detour comes from the hospital. The interventions the hospital recommended to us, like acupuncture, after doing it, my child showed emotional problems that he/she never had before. Therefore, what I mean is that in addition to those interventions recommended by the hospital, we should also let the parents know more about autism and what other interventions are available; otherwise, they will only trust the hospital since they have no idea about other available interventions* (P11, 1C).

*Once my child was diagnosed with autism, the doctor said we should look for interventions. While I have learned more about autism, I still get confused every day. Many parents of autistic children take their children to do interventions like electric shock therapy, transcranial magnetic stimulation, and so on. I am skeptical of these methods after I know more about them. Now if anyone recommends my child to receive transcranial magnetic stimulation, I’m sure I’ll scold him. Therefore, it’s essential to have a guide for parents on what works, what doesn’t, what’s harmful, and so on* (P14, 1C).

**Theme 2 – Behavioral Principles and Techniques.** Many participants expressed an interest in acquiring a foundational understanding of behavior principles due to the accumulated evidence of ABA-based interventions with autistic populations and their popularity in China. The prevalence of interventions rooted in ABA was noted among the participants, with some participants sharing positive experiences with ABA-based services. However, despite the increasing popularity of ABA in China, many participants admitted to having limited comprehension of the science behind these interventions. They desired to learn more about the fundamental principles underpinning ABA strategies and their effectiveness.

*The science behind ABA, right? I want to know what science it is. I would like to know this because I hear about ABA all the time when my child receives the intervention, but I do not know clearly what ABA is* (P03, 1A).

*I was resistant to ABA at first. It might not sound good, but I must say that ABA methods, such as discrete trial training, which we used in class, made me feel like we were training animals. Indeed, many parents think this way: I give the instructions, the child gets a reinforcer from me after completing the tasks, and then I pause for a while and start the next round* (P04, 1B).

Furthermore, participants articulated a specific interest in acquiring knowledge concerning various behavioral techniques and their application within home and community settings. Many participants shared that they actively provide supplementary learning sessions for their children after they come home from different service providers. However, participants also shared that they frequently encounter challenges navigating the intricacies of specific behavioral intervention techniques. Participants needed a comprehensive understanding of behavioral intervention procedures such as reinforcement, generalization, defining a target behavior, and practical guidance on effectively applying behavioral strategies in real-world caregiving scenarios.

*I felt I understood the concepts but did not know how to use them practically. Like the generalization, I think it is hard, and I am unsure if I can help my child generalize one skill to daily living…. I am confused about to which degree the training goal was reached; I remember it was 80% accuracy, and then we could say that the target had been reached. However, there is still a difference when applying those skills to daily life* (P09, 1B).

**Theme 3 – Caregiver Mental Health Support.** Besides the desire to learn to support their autistic children, participating caregivers expressed support needs for themselves, specifically mental health support. Participants shared their experiences of grappling with cumulative anxiety stemming from the diagnosis of autism and the slow developmental progress of their children, even with interventions, exacerbated by expectations that proved to be unrealistic for their unique circumstances. The identified need for mental health support encompassed elements such as guiding new caregivers in accepting the diagnosis, assisting caregivers in establishing realistic expectations for their children, providing strategies for self-care, and fostering connections with other caregivers. Participants emphasized that mental health support for caregivers is pivotal not only for the well-being of the caregivers themselves but also for a healthy family relationship and fostering the positive development of their autistic children.

*As a parent who has gone through this, I feel that you need to add something about how to relieve the anxiety of parents. Although I am at a different stage now, if the child has not been progressing, the anxiety of parents is a lot. Our children will be different from others, which makes us more anxious. If the child is continuously making progress, this anxiety will be offset. However, even typically developing children will encounter obstacles to learning. Whenever our children have difficulties learning, parents of these children, including the parents around me, will be generally anxious and even have anxiety bursts. In most situations, such anxiety will affect the child. So, if you are going to teach parents, my suggestion is to add a module about helping parents relieve anxiety as well as stop self-blame. Until now, I still have the thought sometimes that I brought my child into the world but did not give him a complete and healthy body* (P01, 1A).

#### Topic 2: What Presentation Method Would You Prefer?.

Notably, during the Topic 1 focus group discussions, participants shared comments related to Topic 2 specifically regarding presentation modality and method in caregiver education programs without prompting. For example, one participant expressed the need to be able to understand the content within their own timeframe and another emphasized the use of diverse examples. We synthesized these comments into aligned themes that emerged from the Topic 2 discussions.

**Theme 1—Accessible Language and Cognitive Load.** Participants highlighted the significance of language accessibility, sharing the difficulties they experienced trying to understand medical terms exacerbated by limited Chinese resources. They emphasized the need for content to be presented in a clear and comprehensible manner. Participants also expressed difficulties in comprehending information that came from English sources, acronyms that were directly used from their original English forms (e.g., ABA, DTT), and overwhelming technical terminology and jargon.

*When I read books or listen to lectures, some terms are difficult to understand. I need to spend more time processing them and making them more direct. I am unsure whether other parents would have the same feeling* (P04, 1B).

The cognitive load associated with educational programs emerged as another focal point concerning the delivery method. One aspect was linked to the duration of programs, with caregivers expressing a preference for structured programs that effectively accommodate their schedules and individualized needs. Several participants recommended avoiding presentations exceeding ten minutes, citing considerations such as childcare responsibilities and the potential for overwhelming cognitive load. However, some participants said it would be acceptable to watch longer videos if the topic was complicated and took more time to explain. Another subtheme regarding considerations for cognitive load among participants pertained to the variation of difficulty in different contents. Participants suggested catering to the individual needs of caregivers at various stages of supporting their autistic children. This included addressing the needs of new parents whose children have recently received a diagnosis and those who already possess basic knowledge of autism. This sub-theme underscores the importance of ensuring that online education programs are not only linguistically accessible and temporally feasible but also allow for self-selection and curation.

*For the duration of videos, I feel just like the videos we watched on TikTok, which takes six or seven minutes for each one, so it should not be more than ten minutes. In this way, the length of time would be better* (P04, 2A).

*If online education targets parents, can you divide the content into three levels based on the parents’ stage—novice, intermediate, and advanced? This way, parents can make corresponding choices based on their needs* (P05, 2B).

**Theme 2 – Case Examples and Modeling.** Participants articulated a pressing need to bridge the gap between theoretical understanding and practical implementation in real-life settings, particularly concerning their autistic children’s unique needs. Consequently, participants underscored the value of incorporating case examples spanning various ‘severities’ of autism, as the case examples can serve as instructive illustrations for caregivers on how to tailor and adapt interventions to suit the individualized requirements of their children. Moreover, participants highlighted the efficacy of modeling as a recommended approach to facilitate comprehension and acquisition of intervention techniques and their application within everyday caregiving contexts.

*Can we analyze the case examples more specifically and see if the target behaviors can be categorized? Because each child has different characteristics, they might present different problems* (P02, 1A).

*As a new parent like me, everything is good when my child is in the intervention center with teachers, but I really cannot cope with my children’s needs at home. Part of the reason is my limited ability, so if there is a video, parents can just learn to follow the modeling to teach kids* (P03, 2A).

## Discussion

The current study qualitatively analyzed focus group discussions of Chinese caregivers of autistic children to create a caregiver education program based on the feedback received. While it is not uncommon for studies to solicit perspectives and feedback from caregivers, a substantial number of these efforts often treat caregiver input as an afterthought (e.g., [19,26]). In this study, we integrated caregivers into the pre-development process of caregiver education materials and attempted to equalize the power imbalance between researchers/professionals and caregivers of autistic children.

Before qualitatively exploring caregivers’ needs through focus group discussions, we conducted FES among participating caregivers to understand their levels of empowerment. Our findings suggested that caregivers in this study demonstrated an overall high level of family empowerment, particularly centered on their autistic children. This aligns with their active participation in this study, contributing to the development of a caregiver education program. The detailed response profiles regarding family empowerment also highlighted the need for caregiver training. For instance, the results revealed that caregivers showed greater consistency in their responses under the Self-Efficacy subscale, reflecting their proactive efforts in advocating for their children. In contrast, responses in the Knowledge subscale showed the most variability, indicating greater differences in caregivers’ understanding of key areas related to supporting their children such as knowledge about organizational systems, relevant laws or legislation, and overall information-seeking skills. This variability might be explained by unequal access to educational resources and training opportunities, underscoring the need for focused caregiver training. This finding aligns well with the purpose of the current study, which seeks caregivers’ input on the content and format of a freely accessible caregiver training program.

### The Content

The three identified themes related to the content of online caregiver education programs serve as a window reflecting not only the needs of participants of the current study but also a preliminary view of services for autistic children and their families. For instance, the need to understand the cause of autism aligns with participants’ experiences regarding barriers to their children receiving an autism diagnosis. The barriers caregivers shared include doctors lacking sufficient knowledge and caregivers’ inadequate understanding of child development and autism. Some participants also mentioned misconceptions about the cause of autism and the predominant medical model of autism [8]. Furthermore, the anecdotal experiences of autistic children receiving interventions lacking sufficient or even any research support align with past research of some caregivers turning to non-evidence-based interventions due to scarce resources [27,28].

Our results underscore the challenge of a shortage of medical professionals offering reliable diagnoses and sharing accurate and up-to-date knowledge about autism with caregivers in some communities [29]. In addition, our results highlight limited trustworthy resources and power dynamics between caregivers and service professionals [4]. For many caregivers of autistic children, reaching out to an expert may not be possible due to limited resources. Moreover, in some communities, the ‘experts’ may also need to develop their competency to support autistic individuals and their families.

Although we thought we had constructed our discussion guide with items that we considered pertinent to caregivers of autistic children, we failed to identify caregiver mental health support as an important topic. Past studies focused on the stress of caregivers of autistic children in some Asian countries where ‘behavioral difference’ can be equated to ‘defiance’ and the responsibility of ensuring the child follows social norms falls on the caregivers [30]. Studies also showed that relieving caregiver stress in Western samples, such as receiving support from family members or friends, was not effective in Chinese caregivers of autistic children [31]. Moreover, Liu and colleagues found that 92.6% of Chinese autistic adults resided with their caregivers compared to approximately 50% of Dutch autistic adults with similar adaptive and cognitive [32]. The emergence of caregiver mental health support from our focus group shows caregivers want support they can use independently. Although caregiver education spans knowledge, information, and practical skills [13], current content seldom includes direct mental support for caregivers. Within most caregiver education, caregivers are only considered in terms of their role as caregivers to an autistic child. Although stress and low self-efficacy in caregivers of autistic children are well-documented in the literature (e.g., [30,33]), caregiver mental health support is often separated from caregiver education. The responsibility society places on child rearing and the caregivers’ cultural beliefs that their child is solely their responsibility can negatively impact Chinese caregivers of autistic children [6]. Caregiver education programs in China should include strategies such as creating communities for caregivers of autistic children [34], mindfulness practices [35], and access to caregiver respite care [36].

### Presentation and delivery

In our study, accessibility of information emerged as a crucial factor for caregivers. More knowledge is becoming available for families with autistic children due to increased governmental and non-profit organizations’ efforts [2,37]. However, challenges persist in translating information from Western sources, evaluating the degree of veracity in current resources, and increasing accessibility in terminology and jargon (e.g., [7,9]). Many participants shared that there were few resources with clear and comprehensible language for caregivers. The findings emphasize the importance of addressing the accessibility of language and the limited use of jargon in caregiver education. This also underscores the need to provide caregivers with definitions and the historical backgrounds of some of the terms from medical (e.g., 自闭症 [Self-enclosure disorder] for autism) or Western origins (e.g., ABA) to increase caregiver self-efficacy.

Another notable finding was that the participants asked for more examples of interventions to use at home. The need for increased modeling and practical hands-on training is similarly emphasized by educators in China [38], where there is an apparent shortage of quality practitioners in autism intervention [4]. In an online education program chosen to mitigate service disparities, person-to-person training is not a feasible option. As such, online modules should include as many everyday examples and video modeling as possible, a discussion board for caregivers to communicate, and routine monitoring of the boards by professionals.

### Limitations and future considerations

This study is not without its limitations. Firstly, we acknowledge that the sample size remains relatively modest. However, we would like to share that this study was conducted amidst the challenges posed by the lockdown during the pandemic in China. Nevertheless, a commendable number of caregivers actively participated in the study, underscoring the eagerness of caregivers to engage in the development of an education program. Future researchers should encompass a more diverse representation of caregivers. This inclusivity could extend to caregivers from various backgrounds, including those living in rural areas, low to mid socioeconomic status, and whose autistic children are at different stages of life (e.g., adolescence, adulthood).

Secondly, the current study focused on an online caregiver education program for Chinese caregivers of young autistic children (i.e., ages 6 and under). As such, the prototype videos were created for an online format, and participants may have limited their feedback to a caregiver education format that focused on online delivery. Future studies could include different types of delivery prototypes, including an in-person group and individual options, to gather a broader scope of caregiver feedback.

## Conclusion

Caregiver education program is a promising approach to empower families with autistic children in China, given the limited resources available and the active role caregivers play in their children’s support system. However, the only way caregiver education can truly be utilized to ameliorate barriers to autism service is when the caregiver education program is relevant, acceptable, and accessible to the community it caters to. As such, it is imperative that researchers include caregivers starting with the pre-development stages, continue to involve caregivers in each stage of development by inviting and incorporating their feedback and considering the systematic and infrastructural issues of the community. Within a community-engaged participatory research framework [39], researchers partner with community participants to identify community needs and utilize lived experiences to target research that is essential to the community. Similarly, when planning caregiver education researchers should elevate caregivers as co-designers to create an education program that specifically caters to the unique needs and specific challenges faced by caregivers of autistic children in respective communities.

## Supporting information

S1 File Topic Guide. (DOCX)

S2 File Positionality Statements. (DOCX)

S3 File Chinese Original Transcripts of Participant Quotes. (DOCX)
