# Supplementary material for: Creating a caregiver education program with chinese caregivers of autistic children: A community engaged process
Source: PLoS One. 2026 Aug 13;21(8):e0355749. doi: 10.1371/journal.pone.0355749 (PMC13472438; doi:10.1371/journal.pone.0355749)
Supplement: Supplementary file 1 [file pone.0355749.s001.docx]

**Supporting Information**

**S1. Topic Guide**

**[Module 1: Introduction to Autism, Evidence-Based Practice and ABA]**

***Part 1: Autism***

- Brief history
- Current knowledge
- Genetics, environment
- Neurodevelopmental aspects
- Neurodiversity, atypical development

***Part 2: Evidence-Based Practices***

- What makes ABA-based interventions so effective for ASD
- Evidence-based practices
- How are they established?
- What are some limitations?

***Part 3: Introduction to ABA***

- What is ABA?
- Foundational concepts of ABA in relation to autism interventions

**[Module 2: Skill Acquisition 1]**

***Part 1: Developmental Progression***

- Developmental milestones and risk signs
- Case Examples or Scenarios of Areas of Developmental Disabilities
  1. Autism
  2. Briefly address the following: EBD, ADHD, Comorbidities

***Part 2: Selecting a Target Skill***

- How to select target skills
  1. Build upon child’s developmental milestones
  2. Address difficulties associated with developmental disabilities
  3. Using assessments
     1. ABC data
  4. Stakeholder involvement
- General considerations
  1. Skill domains (e.g., social, language, communication, adaptive, pre-academic)
  2. Operational definition
     1. SMART Goals
  3. How to facilitate skill acquisition from an A-B-C perspective
- Putting them together: A case study
  1. Social communication

**[Module 3: Reinforcement]**

***Part 1: General Overview***

- Definition of reinforcement
- Understanding the difference between reinforcement and bribing
  1. Major misconceptions related to ABA and interventions related to behavior change.
- What are all of the ways reinforcement may look like?
  1. Examples (e.g., simple praise, high fives, playing a game)
- Recommended reinforcement ratio → 4:1
  1. Emphasis on ‘catching your child being good/having good behavior’ and being cognizant of this instead of reprimanding
- Pairing
  1. Ensuring that the individual who is implementing the intervention work is also pairing with the child’s preferred reinforcers before placing heavy demands of the intervention on the child
  2. Taking the time to develop associations with the interventionist/therapist/parent and reinforcers, discussing this as a practitioner strategy to build rapport

***Part 2: Positive Reinforcement and Negative Reinforcement***

- Positive reinforcement
  1. Definition
  2. Examples
  3. Deliver reinforcement when child engages in target goal
  4. Social reinforcement (praise)
- Negative reinforcement
  1. Definition
  2. Examples (inadvertently reinforcing behaviors not intending to reinforce)
  3. Cue child to use target skill or behavior
  4. Remove negative reinforcer when target skill or behavior is used
  5. Transition to positive reinforcement

***Part 3: Preference Assessment***

- Types of reinforcers
  1. Why doing a preference assessment is so important
     1. Expanding options for reinforcement
     2. Satiation
- Types of preference assessments
  1. Observation of students with measurement of engagement with items
  2. Example in the natural environment
  3. Paired choice: chooses 1 of 2 items presented in pairs
  4. Example in the natural environment
  5. Contrived
- Data collection for preference assessments
  1. Did it actually work?
- Preferences change; assess often
  1. Satiation: too much of a reinforcer
  2. What is motivating today, may not be tomorrow
  3. Prevention techniques:
  4. Menu of reinforcers: top 5-10 choices
  5. Introduction of novel reinforcers throughout

***Part 4: Things that Affect Reinforcers***

- Setting events
  1. Examples
- Establishing operations
  1. Examples
- Abolishing operations
  1. Examples

***Part 5: Token System***

- Preparing materials
- Components of the token economy program
  1. Target skill or behavior needed to perform
  2. Set token objectives
  3. Provide a token
  4. Describe why they earned a token
  5. Learners select reinforcement (menu)
  6. Thin tokens and use consistently across settings

***Part 6: Reinforcement Thinning and generalization***

- Adjustment reinforcement based on child meeting performance criteria
- Ensure use across people and setting
- Self-reinforcement: Self-monitoring
  1. Video examples: APPS

**[Module 4: Skill Acquisition 2]**

***Part 1: Embedded DTT***

- What it looks like in a clinical setting/home
- Video example

***Part 2: Naturalistic teaching (Brief. Separate Module Follows)***

- Incidental teaching

***Part 3: Chaining/task analysis***

- Video example (e.g., handwashing, toothbrushing)
- Example task analysis

***Part 4: Shaping***

***Part 5: Prompting***

- Prompt hierarchies
- Delayed prompting
- Graduated delayed prompting
- Fading

***Part 6: Video Based Interventions***

***Part 7: Generalization***

- Appropriate vs. inappropriate examples
- Discrimination
  1. Discrimination skills utility
- Stimulus control transfer
  1. Example

**[Module 5: Naturalistic Developmental Behavioral Interventions]**

***Part 1: Role of Development in Autism Interventions***

- Refer to Module 2 → re: developmental milestones

***Part 2: Benefits of this Evidence-based Practice***

- Building Relationships
  1. Refer back to Module 3

***Part 3: Natural Environment***

- What it is

***Part 4: Routine-based interventions***

- Where would teaching be embedded in natural contexts?
  1. Mealtime, playtime, with siblings, etc.
- Common Elements
  1. Environmental arrangement
  2. Face-to-face and on the child’s level
  3. Following your Child’s Lead
  4. Positive affect and animation
  5. modeling appropriate language
  6. Responding to attempts to communicate
  7. Using communicative temptations
  8. Frequency of direct teaching episode
- Quality of direct teaching episode
  1. Refer back to Module 4
- Natural and contingent reinforcement

***Part 5: Manuals***

- Examples of NDBI Models
  1. Referring to NDBI under the ABA umbrella
     1. (referring back to module 1),
  2. Then NDBI being another umbrella
- Help is in your hands → parent and clinician resource
- Project ImPACT
  1. Parent ImPACT parent resource
- PRT
- EMT
- JASPER
- Social ABCs
- Early Achievements

***Part 6: Strategies that Enhance Motivation***

- Refer back to Module 3
- Refer to and give examples from Table 6.1 from NDBI text?
- Considerations for Goal Selection
  1. Selecting Goals with examples/case studies
  2. Refer back to Module 2, providing more examples in the following areas:
     1. Language Goal
     2. Social Skills
     3. Play
     4. Adaptive Behaviors

***Part 7: Considerations for Data Collection in NDBI***

- Caregiver Involvement in Data Collection
  1. Refer to Module 6

**[Module 6: Behavior Reduction]**

***Part 1: Behaviors Typically Targeted***

- Tantrums
- Aggression
- Self-injury
- Echolalia/Repetitive behaviors
  1. That interfere with learning/daily routines
- Sensitivity to self-stimulatory behaviors that serve a function

***Part 2: Assessment of Problem Behavior***

- Is the frequency high enough, does it interfere with daily activities?
- Functions of Behavior
  1. Functional Analysis
  2. Functional Assessment

***Part 3: What is a Behavior Intervention Plan?***

- Components of BIP
- ABCs of Behavior for Behaviors Targeted for Reduction
- Antecedent Based interventions
  1. Avoiding triggers
  2. Daily Routines
  3. Timers
- Functional Communication Training
- Consequent Based Interventions
  1. Schedules of reinforcement
  2. Token Systems
