# Supplementary material for: Creating a caregiver education program with chinese caregivers of autistic children: A community engaged process
Source: PLoS One. 2026 Aug 13;21(8):e0355749. doi: 10.1371/journal.pone.0355749 (PMC13472438; doi:10.1371/journal.pone.0355749)
Supplement: Supplementary file 3 [file pone.0355749.s003.docx]

**Supporting Information**

**S3. Chinese Original Transcripts of Participant Quotes**

**Topic 1: What Content Would You Like to See in a Caregiver Education Program?**

***Theme 1 - Autism: From Cause to Intervention***

We first want to know the cause of these children’s symptoms. In other words, maybe the priority is to find out the reason. Figuring out what is affecting the child and what scientific methods are appropriate for intervention. I think this might be a better way for families and parents (P03, 1A). 我们首先想了解的就是小朋友表现出来的一些现象是什么原因导致的，就是说可能一开始要先找出这个原因，他是哪一方面受到的影响和表现出来这些东西，然后适合用什么样的科学的、你们的科学方法来干预，我觉得这样可能比较好一点。对家庭，对家长而言。

My child was diagnosed at the age of three, but the diagnosis at first was very unclear. We spent two to three hours on the diagnostic process, filling in measures, etc. The doctor only took 10 minutes to observe my child and said that my child is autistic and needs interventions. I was very confused and skeptical about the diagnosis - how the doctor could only use a few minutes to diagnose my child. After I went back home, I looked up the information about autism and did observations on my child, and finally accepted this diagnosis (P13, 1C). 然后呃我小孩子三岁多的时候是确诊的，但是到底是这个（诊断）是他没有确切的，也不清楚。然后花了两三个钟去弄，他 （医生）看小孩子只是十分钟。然后我感觉这些东西。不包括很多家长可能都会出现这个问题，就是说小孩子一进去了之后，没没谈个多久都是这样子的。那会儿我就进去填了做了三张表，然后进去跟医生，大概主治医生聊了大概，他也就看孩子，也就看了几分钟。就跟我说，哦自闭，去做干预。所以说我对这个诊断当时也是有一点模棱两可的，你说几分钟就开始看出孩子自闭了？呃，但是通过我在家里头也观察嘛，后来我就自己上网找一些这些方面的知识，所以说后来我一条一条一条一条看，才最终也是说服我自己。

Parents who just received their child’s diagnosis do take the wrong path, and I think the first detour comes from the hospital. The interventions the hospital recommended to us, like acupuncture, after doing it, my child showed emotional problems that he/she never had before. Therefore, what I mean is that in addition to those interventions recommended by the hospital, we should also let the parents know more about autism and what other interventions are available; otherwise, they will only trust the hospital since they have no idea about other available interventions (P11, 1C). 就前期新家长啊，确实是一定会走弯路，我觉得第一个弯路我也感觉是医院给我们的方法，那些什么啊包括那个针灸。针灸是医院里面推给你的做了之后，孩子反而情绪这方面出来，以前没有。除了医院之外，我们还可以给新家长普及一下干预方法，让家长多了解一下。不然的话新家长真的是只是信任医院哦，因为新家长他真的不知道还有其他路的。

Once my child was diagnosed with autism, the doctor said we should look for interventions. While I’ve learned more about autism, I still get confused every day. Many parents of autistic children with autism take their children to do interventions like electric shock therapy, transcranial magnetic stimulation, and so on. I'm skeptical of these methods after I know more about them. Now if anyone recommends my child to receive transcranial magnetic stimulation, I'm sure I'll scold him. Therefore, it's essential to have a guide for parents on what works, what doesn't, what's harmful, and so on (P14, 1C). 孩子确认了自闭症以后，医生说自闭症了然后就说要找方法，怎么干预嘛。然后就我现在每天也去逛知乎，因为关注了这些问题，自闭症的问题，他就每天都有问题问到我。 好多问题就，好多孩子已经确诊，以后家长带孩子做一些电疗啦，什么经颅磁啊。那些东西我现在了解以后我是对这些方法是持怀疑态度的。因为像我现在对经颅磁肯定是谁跟我说这要我孩子做这个，我肯定是会会骂他两句的。嗯。对，因为所以说所以说我觉得就是说对哪些方法有用，或者哪些方法没用，哪些方式还是有害的，会对孩子有益的这些对我们家长要有一个指导。

***Theme 2 - Behavioral Principles and Techniques***

The science behind ABA, right, I want to know what science it is. I would like to know this because I hear about ABA all the time when my child receives the intervention, but I don’t know clearly what ABA is (P03, 1A).  ABA的那个背后的科学吧，想了解一下它是怎样的一门科学。我觉得这个对于我来说我比较想知道，因为小孩在干预过程中都一直在说ABA、ABA的，但是具体好像不是很清楚。

I was resistant to ABA at first. It might not sound good, but I must say that ABA methods, such as discrete trial training which we used in class, made me feel like we were training animals. Indeed, many parents think this way: I give the instructions, the child gets reinforcer from me after completing the tasks; then I pause for a while and start the next round. However, I am learning about ABA and how it was delivered in classes, and I have to admit that ABA is effective. The methods can be learned and applied easily, and we can see whether teaching our kids with ABA is effective or not (P04, 1B). 就是我觉得吧，其实我一开始。比较抵触这一个教学方法的。就是说句不太好听的，就感觉它像我们上课比较多用那种DTT的教学模式，它会让你感觉好像就是训练动物也是这种操作。是可能真的是确实会有很多家长会这么想诶，我发出一个指令，你完成我的指令了，然后我强化你这个对的行为，给你奖励，我们再停顿一下，进行下一个回合。但是我在慢慢的就是这个学习啊，上课什么的过程，但是确实这个用于我们的小孩身上很有效。

I felt that I understood the concepts but didn't know how to use them in practical settings. Like the generalization, I think it is hard, and I am unsure if I can help my child generalize one skill to daily living…. I am confused about to which degree the training goal was reached; I remember it was 80% accuracy, and then we could say that the target had been reached. However, there is still a difference when applying those skills to daily life (P09, 1B). 好像我感觉概念我都能理解，就是，但是不知道实际操作的时候比较难。我就不知道我到时候实际，能不能说引导那个小孩子能够泛化到生活上面去。......一个项目到达到什么一定程度呃，原来好像说是那个正确率是达到百分之八十嘛，然后就说这个项目就可以过了。可是实际上的话，我觉得说好像这种的话，你应用到生活中还是有差距嘛。

***Theme 3 - Caregiver Mental Health Support***

As a parent who has gone through this, I feel that you need to add something about how to relieve the anxiety of parents. Although I am at a different stage now, if the child has not been progressing, the anxiety of parents is a lot. Our children will be different from others, which already makes us more anxious. If the child is continuously making progress, this anxiety will be offset. But in fact, even typically developing children will encounter obstacles to learning. Whenever our children have difficulties learning, parents of these children, including the parents around me, will be generally anxious and even have anxiety bursts. In most situations, such anxiety will affect the child. So if you are going to teach parents, my suggestion is to add a module about helping parents to relieve anxiety as well as stop self-blame. Because until now, I still have the thought sometimes that I brought my child into the world but did not give him a complete and healthy body (P01, 1A). 如果只是培训家长的话，你们需要另外增加一下，怎么样缓解家长的焦虑。因为我是过来人，我在不同的阶段，如果孩子一直没进步，我的焦虑感，因为毕竟我们我们这种孩子会和其他孩子也不一样的，我们本身这里面这种焦虑感就很多了。如果孩子一直在持续的进步，这种焦虑感会抵消。但是实际上，但是哪怕是普通孩子学习也会遇到障碍，每当遇到障碍的时候，这就是我们家长，就是普遍，包括我身边的家长都会普遍焦虑，焦虑爆表。而在大多数存在这种情况下会影响到孩子。所以说刚才你说如果是教家长的话，我的建议就是要着重有一章就是怎么去缓解家长焦虑以及自责的心理。因为一直到现在，我我有时候我也我也会有这种感觉，就是我把我的孩子带到这个世上，并没有给他一个完整健康的身体啊。

**Topic 2: What Presentation Method Would You Prefer?**

***Theme 1 - Accessible Language and Cognitive Load.***

When I read books or listen to lectures, I think it’s hard to understand some terminologies. I need to spend more time processing them and changing them into a more direct way to understand them. I am not sure whether other parents would have the same feeling (P04, 1B). 我对于这种比较专业的术语很难理解。要花时间去把它想象成比较直白一点的表达方式，我不知道别的家长会不会。

For the duration of videos, I feel just like the videos we watched on TikTok, which basically takes six or seven minutes for each one, so it shouldn't be more than ten minutes. In this way, the length of time would be better (P04, 2A).如果就是干货多一点的话，时长都是无所谓的，就像你讲一个事情讲的多一点的话也无所谓。那时长啊，时长十多分钟都无所谓再多一点都无所谓。嗯，能讲清楚。

If online education targets parents, can you divide the content into three levels based on the stage the parents are - novice level, intermediate level, and advanced level? In this way, parents can make corresponding choices based on their needs (P05, 2B). 如果咱这个视频是针对线上家长的，您看能不能分成就是这是初、中、高，三个阶段? 这样对于这样分分级之后分成初、中、高三个阶层之后家长进行对应的选择。

***Theme 2 - Case Examples and Modeling.***

Can we analyze the case examples more specifically and see if the target behaviors can be categorized? Because each child has different characteristics, they might present different problems (P02, 1A). 就是我感觉这个我们后期能不能就说拿那个案例来具体分析，然后看一下能不能他这种是不是有归类的，就说他这种出现这个问题，是不是有就是有归类这种的，就是类似这种意思..... 因为就是每个小孩子，他性格不一样嘛，他反馈出来的那个东西也不一样，所以的话真的不知道怎么搞。

As a new parent like me, everything is good when my child is in the intervention center with teachers, but I really cannot cope with the needs of my children at home. Part of the reason is my limited ability, so if there is a video, parents can just learn to follow the modeling to teach kids (P03, 2A). 我们这种就比较新的家长，在机构，老师教的很好，可是在回家，我感觉我们真的是就应付不了他，就是可能也是能力的原因就是多多一点这种的，然后也让一些家长可以按照一下，就是起码有个视频，然后可以按照上面的模式，然后去教一下小孩子。
